# Ultrasound-guided pulsed radiofrequency treatment for distal suprascapular neuropathy: A case report

**DOI:** 10.1097/MD.0000000000022469

**Published:** 2020-09-25

**Authors:** Yang-Hoon Chung, Joon-Ho Lee, Bon-Sung Koo, Jaewoong Jung, So Jeong Lee

**Affiliations:** Department of Anesthesiology and Pain Medicine, Soonchunhyang University Bucheon Hospital, Soonchunhyang University College of Medicine, Bucheon, Republic of Korea.

**Keywords:** brachial plexus block, case report, nerve block, pulsed radiofrequency treatment, rotator cuff injuries, shoulder pain, ultrasonography

## Abstract

**Rationale:**

Suprascapular neuropathy is a rare cause of shoulder pain, and patients usually presents with posterosuperior shoulder pain and weakness on forward flexion and external rotation. Suprascapular neuropathy associated with rotator cuff pathology has received attention as an emerging cause of this condition. Suprascapular nerve (SSN) block can be used in these patients, and pulsed radio frequency (PRF) can be applied to achieve a long-term effect. Several studies have reported on PRF treatment of the SSN for shoulder pain, but most applied treatment to the nerve trunk under the transverse scapular ligament. This report describes a patient with suprascapular neuropathy treated with selective application of PRF to the distal SSN under ultrasound guidance.

**Patient concerns:**

A 68-year-old woman suffered from right posterior shoulder pain after traumatic full thickness rotator cuff tear. Her pain was not diminished despite of 2 surgeries.

**Diagnoses:**

She was diagnosed with entrapment of the distal SSN in the spino-glenoid (SGN) notch and suprascapular neuropathy.

**Interventions:**

She underwent surgery to decompress the entrapped SSN in the SGN. After that, we applied PRF on the distal SSN under ultrasound guidance for persistent pain. This treatment was repeated 3 times.

**Outcomes:**

PRF treatment resulted in a slight reduction in the visual analogue scale (VAS) pain score from 7–8/10 to 5–6/10 at the 2 weeks follow-up, and to 2–3/10 at the 1 month follow-up. The reduction in pain was maintained at the 1 year follow-up.

**Lessons:**

PRF treatment of the SSN is typically approached from the main branch in the suprascapular notch. We selectively applied PRF to the distal SSN close to the SGN. This technique was safe and effective.

## 1 Introduction

The suprascapular nerve (SSN) provides sensory and motor innervation to a large portion of the shoulder, usually originating from the fifth and sixth cervical nerves. The SSN is vulnerable to injury at several sites as it passes from the neck to the shoulder.^[1]^ Among various reasons for injury, compression or traction of the nerve in association with rotator cuff pathology is a likely cause of suprascapular neuropathy.^[2]^

Nonoperative treatment is the first choice for most patients with suprascapular neuropathy. Similar to several other neuropathies, pulsed radio frequency (PRF) is an emerging technique for treating this kind of neuropathy when other conservative treatments are ineffective. Although the clinical evidence is limited regarding the effect PRF on peripheral nerves, several reports indicate that PRF of the SSN successfully relieves shoulder pain.^[3–5]^ However, PRF treatment of the SSN is typically approached from the main branch in the suprascapular notch. The introduction of ultrasound-guided nerve block techniques has enabled a more selective approach, targeting the peripheral nerves and surrounding structures, and can also be applied to the SSN.^[6,7]^

We report successful use of ultrasound-guided PRF treatment of the distal SSN for intractable posterior shoulder pain due to suprascapular entrapment neuropathy.

## 2 Case report

**Figure 1:**
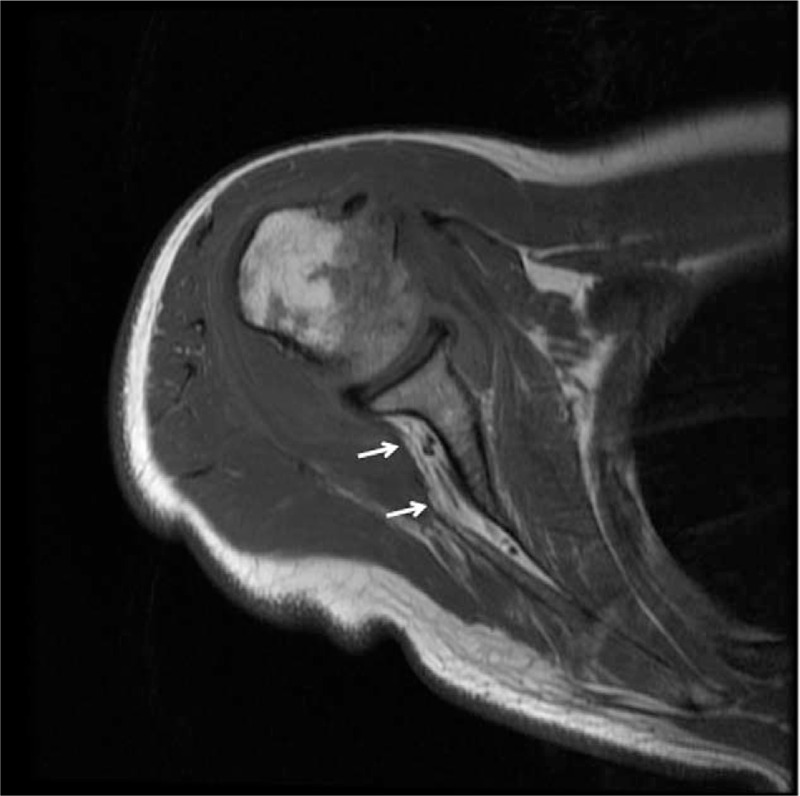
Axial T2-weighted magnetic resonance images of the right shoulder demonstrating fatty atrophy of the infraspinatus muscle (arrows).

**Figure 2:**
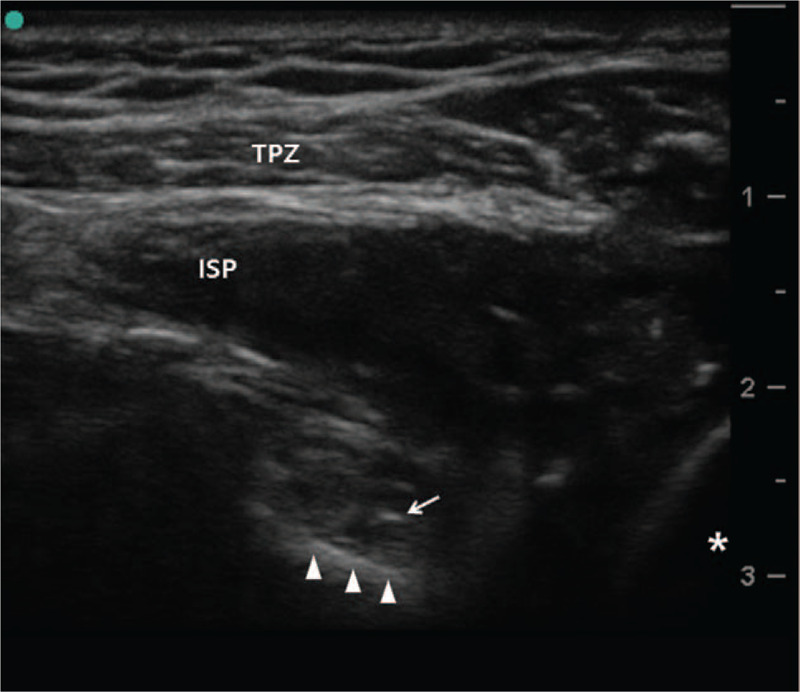
Ultrasound imaging of applying radiofrequency probe on suprascapular nerve in the spinoglenoid notch. Arrow indicates the needle tip and arrow heads indicate spinoglenoid notch near the glenoid (asterisk). ISP = infraspinatus muscle, TPZ = trapezius muscle.

A 68-year-old woman was referred to our clinic complaining of electric shock-like pain (visual analogue scale [VAS] pain score of 7–8/10) in the right posterior shoulder. She had no remarkable past medical history. She underwent arthroscopic surgery for a full thickness rotator cuff tear about 4 months previously. After surgery, pain on abduction of the shoulder was mostly relieved but she still complained of posterior shoulder pain at rest. No difference in pain intensity was observed between rest and during movement. Preoperative magnetic resonance imaging showed atrophic signal changes in the infraspinatus muscle (Fig. 1). She was referred to the department of orthopedic surgery to confirm whether entrapment of the distal SSN in the spino-glenoid notch (SGN) was the source of her pain. Although electromyography and nerve conduction velocity studies revealed lesions of the distal SSN, the surgeon wanted to confirm the lesions by nerve block at the SGN before surgery. The distal SNN was blocked under ultrasound with a 6 to 13 MHz linear array transducer (SonoSite S-nerve; SonoSite Inc., Bothell, WA) using the out-of-plane technique at the SGN with 1 mL of 0.25% ropivacaine (Fig. 2).^[6]^ The nerve block relieved the pain temporarily (VAS pains score dropped to 2–3/10). She was diagnosed with entrapment of the distal SSN in the SGN and suprascapular neuropathy.

She underwent a second surgery to decompress the entrapped SSN in the SGN 1 week later. However, the pain was not diminished at all after surgery. Her medications at that time included, pregabalin, OxyContin/naloxone, and acetaminophen/tramadol; the dosages were adjusted but the pain persisted. At the second visit to our clinic, SSN block was performed 3 times, as described above, with 2 mL 0.25% ropivacaine and 10 mg triamcinolone with a 1 week interval. However, her pain was relieved for only 2 and 3 days. We decided to apply PRF on the distal SSN. The PRF was done with a radiofrequency lesion generator (Diros OWL URF-3AP; Diros Technology Inc., Markham, ON, Canada) using a straight radio frequency probe with a 5 cm long, 5 mm active tip (467–005-TCH-S). After applying local anesthesia to the skin, the cannula was inserted under ultrasound guidance close to the SGN. After confirming the suprascapular artery near the nerve, the cannula was advanced carefully, so as not to damage the nearby vessels, until paresthesia was reported at 50 Hz and 0.3 to 0.5 mA. The motor response was also checked with 2 Hz of stimulation. PRF was applied for 150 seconds at 45 V and a pulse duration of 20 ms, not exceeding 42 °C. This treatment was repeated 3 times and resulted in a slight reduction in the VAS pain score from 7–8/10 to 5–6/10 at the 2 weeks follow-up, and to 2–3/10 at the 1 month follow-up. The reduction in pain was maintained at the 1 year follow-up. She was satisfied with her conditions. She underwent a rehabilitation program to restore shoulder function. Written informed consent was obtained from the patient for publication of this case report and accompanying images

## 3 Discussion

Suprascapular neuropathy is a rare cause of shoulder pain and usually occurs due to compression or traction following trauma, space-occupying lesions, or repetitive overhead activities.^[1,8]^ SSN traction in the suprascapular notch or SGN after a massive rotator cuff tear is another cause of suprascapular neuropathy and constitutes a significant part of this condition.^[2,9,10]^ Patients typically present with posterosuperior shoulder pain and weakness on forward flexion and external rotation. Atrophy of the supraspinatus or infraspinatus muscles is often detected during inspection. Electrodiagnostic testing, magnetic resonance imaging, and selective nerve block are useful diagnostic tools.^[11]^ Nonoperative treatments, including nonsteroidal anti-inflammatory drugs, physical therapy, and nerve blocks, are the typical first-choice treatments and surgery is offered if these treatments fail. However, there have been few established indications for surgery in patients with isolated suprascapular neuropathy related to rotator cuff tears until now. Furthermore, similar to other musculoskeletal disorders, there are few alternative treatment modalities available if surgical treatments fail.^[1,2,8,10]^ The natural history of this condition is not well known, so there is no recommended duration for non-surgical treatments, except when space-occupying lesions exist.^[11]^

SSN blocks have been used to manage various kinds of pain around the shoulder.^[12]^ They can also be used to confirm the exact cause of pain when the results of electrodiagnostic testing are inconclusive. The most commonly used techniques involve superior and posterior approaches. Regardless of the technique, the majority of nerve block methods target the SSN at the suprascapular notch under the transverse scapular ligament.^[12]^ Messina et al^[6]^ suggested approaching to the distal SSN nearby to the SGN. The efficacy of this technique has not yet been validated, but it can be useful in certain circumstances.

Applications of PRF to treat peripheral nerves have been increasing. Clinical evidence of the efficacy of PRF for treating pain originating from peripheral nerves remains limited, but several reports show the effectiveness of PRF on the SSN.^[3–5]^ However, even though PRF is a relatively safe procedure compared with conventional heat radio frequency, it is invasive and can affect cellular structures.^[13,14]^ Thus, a more selective approach to the peripheral nerve could make this technique safer and more effective, and high resolution ultrasound provides additional guidance.

In conclusion, we report our experience of selectively applying PRF to the distal SSN to treat posterior shoulder pain due to suprascapular neuropathy.

## Author contributions

**Conceptualization**: Yang-Hoon Chung, Joon-Ho Lee.

**Investigation:** Bon-Sung Koo, Jaewoong Jung.

**Methodology:** So Jeong Lee.

**Resources:** Bon-Sung Koo, Jaewoong Jung, So Jeong Lee.

**Visualization:** Yang-Hoon Chung.

**Writing – original draft:** Yang-Hoon Chung, Joon-Ho Lee.

**Writing – review & editing:** Yang-Hoon Chung, Joon-Ho Lee, Bon-Sung Koo, Jaewoong Jung, So Jeong Lee.
